# DFT Calculations of Structure and IR Spectra of M@C_60_ and M_2_@C_60_ Endofullerenes (M=Sc and Y)

**DOI:** 10.3390/molecules30163421

**Published:** 2025-08-19

**Authors:** Alexey V. Krisilov, Igor V. Nechaev, Vladislav E. Chernov, Gie Eli Kallu

**Affiliations:** 1 Faculty of Physics, Voronezh State University, 394018 Voronezh, Russia; alexph@mail.ru; 2 Faculty of Chemistry, Voronezh State University, 394018 Voronezh, Russia; nechaev_iv@chem.vsu.ru (I.V.N.); kallou1993@gmail.com (G.E.K.)

**Keywords:** endohedral metallofullerenes, lanthanides, IR spectra

## Abstract

The endohedral metallofullerenes with a rare-earth metal encapsulated into the carbon cage are nanoparticles with potentially wide applications. We present the results of our quantum-chemical modelling of Sc@$C_{60}$, Y@$C_{60}$ and Sc_2_@$C_{60}$, Y_2_@$C_{60}$ endofullerenes and calculate their structures and vibrational spectra. Our calculations show that the encapsulation of an additional metal atom inside the carbon cage significantly changes the vibrational spectrum of endofullerene. The most significant changes in the far-IR (below 600 cm^−1^) spectra are due to the metal–carbon cage vibration modes.

## 1. Introduction

The unique properties of fullerenes offer a wide range of opportunities for fabricating various single-molecule electronic devices [1]. An endohedral fullerene (or endofullerene) is a carbon shell of fullerene C_60_ or higher fullerenes such as C_70_, C_78_ or C_82_, within which one or more atoms are encapsulated [2,3]. Endohedral metallofullerenes (EMFs) have attracted considerable attention due to their unique properties and wide range of possible applications [4,5,6,7,8,9,10,11]. In addition to their unique electronic properties, endofullerenes have enhanced chemical stability compared to their non-metal-enriched counterparts [12]. EMFs are characterized by large light absorption intensity in the visible and IR ranges, which leads to increased photocurrent and photovoltaic device efficiency [13]. EMFs are charge transfer complexes because they combine both electron donor and electron acceptor in one molecule [14,15]. This property is very important for the operation of photovoltaic systems, where rapid separation of electron–hole pairs is required to prevent recombination and ensure efficient energy conversion. By choosing the type and number of metal atoms embedded in the fullerene, the width of the forbidden band gap can be adjusted, which can optimize the absorption spectrum and increase efficiency of energy conversion in photovoltaic systems [16,17]. Controlling the properties of EMFs by selecting suitable metal atoms and functionalizing the fullerene shell offers great potential for the development of new materials, electronic and optoelectronic devices, including semiconductors, photodetectors, and transistors [18,19,20,21,22].

The geometrical and electronic structure as well as spin properties of scandium and yttrium dimetallofullerenes (di-EMFs) are being actively studied [23,24]. Some EMFs containing scandium and yttrium atoms are chemically stable molecular nanomagnets, which are also known as single-molecule magnets [25]. Structures possessing magnetic bistability can be used to store magnetic information at the molecular level, which attracts researchers in the field of quantum information technology and the development of high-density information storage. Studies of the electronic structure, magnetic interactions and molecular vibrations of EMFs, as well as their dependence on the spin state are of particular interest [26,27]. Scandium and yttrium EMFs also exhibit high efficiency in photovoltaic applications and offer new opportunities to enhance the performance of various optoelectronic devices [28,29,30].

DFT calculations are widely used for modelling the endohedral fullerenes, both with a single atom [31] or a molecule [32] encapsulated inside the carbon cage. In our previous works (see, e.g., [33,34] and references therein) we conducted DFT analysis to compare the structures, symmetries, spins and dipole moments of lanthanide EMFs by means of quantum-chemical calculations and obtained their vibrational spectra in different spin states. In this work, we calculate the stable structures and IR spectra of the mono- and di-EMFs of scandium and yttrium using the density functional method (DFT).

It should be noted that the EMFs of rare-earth metals have already been extracted and studied experimentally. For instance, Ref. [35] studied the La@C_60_ and Gd@C_60_ EMFs and the $\mathrm{Gd}@C_{60}{\left({\mathrm{CF}}_{3}\right)}_{3}$, $\mathrm{Gd}@C_{60}{\left({\mathrm{CF}}_{3}\right)}_{5}$ and $\mathrm{La}@C_{60}{\left({\mathrm{CF}}_{3}\right)}_{5}$ functionalized EMFs. Refs. [36,37,38,39,40] report on synthesis of EMFs with C_60_ that encapsulate Y, La, Ce, Pr, Nd, Gd atoms. The extraction of these EMFs was performed using aromatic solvents such as pyridine and aniline, the isolation of pure M@C_60_ EMFs being significantly complicated due to the formation of exohedral adducts with solvent molecules.

During the synthesis of EMFs, structures can be formed with both metal clusters M_x_@C_2n+2_ and metal carbides inside the carbon cage (M_x_C_2_)@C_2n_. Determining whether a carbide or a metal cluster is inside a fullerene has been an important issue since 2001, when metal-carbide EMFs (Sc_2_C_2_)@C_84_ were first isolated [41], up to recent studies of the $Y_{2}@C_{84}$ and $Y_{2}C_{2}@C_{82}$ [42], ${\mathrm{Sc}}_{2}@C_{70}$ and ${\mathrm{Sc}}_{2}@C_{68}$ [43], ${\mathrm{Sc}}_{2}C_{2}@C_{80}$ and ${\mathrm{Sc}}_{2}@C_{82}$ [44], or ${\mathrm{Er}}_{2}@C_{80}$ and ${\mathrm{Er}}_{2}C_{2}@C_{78}$ [45].

Di-EMFs M_2_@C_80_ (M = Sc, Y, Gd, Er, Lu) have a triplet electronic state due to the half-filled low-lying binding orbital of the metal dimer (one unpaired electron is transferred from this orbital to the carbon cage). Such high spin states makes di-EMFs promising candidates for molecular qubits or spin-filters. IR-active metal–cage and metal–metal vibrational modes couple to these spin states via spin–phonon interactions, directly influencing decoherence times. This makes di-EMFs the simplest organometallic compounds with M–M bonds which possess a unique molecular magnetism [46]. Encapsulating the second rare-earth atom into the carbonic cage allows to form single-electron metal–metal bonds which can enhance magnetic coupling between the atoms and create a better-performing single-molecular magnet [27].

In addition, di-EMFs are unique objects in which metal atoms are isolated from the external environment by a carbon cage, which is of interest from the point of view of analyzing the nature of the bond between two metal atoms and studying the influence of additional non-metal atoms inside the fullerene on the stability of the molecular structure [47,48].

Although Y@C_60_ has already been obtained during synthesis [49], to date, the Y_2_@C_60_ di-EMF has not been isolated so far in the synthesis of EMFs. The same is the case for mono- and di-EMFs of scandium with C_60_ cage. It should be noted that structures and isomers not identified in the first synthesis of endofullerenes of the corresponding metal can sometimes be obtained decades later [50]. Therefore, the calculation of IR spectra of the above EMFs can contribute to their detection and identification.

Raman spectroscopy should be mentioned as a valuable technique to use for the identification of molecules using intuitive parameters obtained from experiments, such as line width, peak intensity, and peak position changes. However, IR spectra are also informative for detection and identification of molecular species. Note for instance that it was IR spectrum calculation that allowed for distinguishing between the ${\mathrm{Sc}}_{2}@C_{84}$ [44] and hypothetical ${\mathrm{Sc}}_{2}C_{2}@C_{82}$ di-EMFs [3].

High-accuracy IR predictions can contribute to a library of vibrational fingerprint searches for EMFs, delivering the essential “spectral coordinates” for detecting and characterizing these species even before their macroscopic isolation has been conducted in the laboratory. This is especially important in the cases when Raman measurements are not available. For example, the growing data recorded by the rapidly developing IR astronomy facilities require knowledge of IR spectra for a large number of molecular species, including organic compounds [51]. EMFs are potential emitters contributing to the observed infrared spectra in fullerene-rich planetary nebulae and circumstellar shells [52] and they can be candidates for explaining several unidentified IR lines. The detection of fullerene derivatives (such as mono- and di-EMFs) in planetary nebulae can enrich our knowledge of the chemical evolution of the Universe.

The experimental frequencies of atom vibrations inside the EMF cage are difficult to measure. The influence of the Rayleigh wing of the scattering of the initial radiation line makes it difficult to detect small frequency shifts. Analysis of far-IR spectra can be complicated by the low energy of photons. Therefore the theoretical calculation of the vibration frequencies of metal atoms in EMFs is an important task.

This paper is organized as follows. The calculation scheme is described in Section 3. The results of the calculations are presented in Section 2. The geometry of the calculated structures is described in Section 2.1, and their stability is shortly discussed in Section 2.2. The general discussion of the vibrational spectra is given in Section 2.3. The IR spectra calculated for both the mono- and di-EMFs consist of many lines, most of which are absent in the IR spectra of empty fullerene C_60_ due to symmetry forbidding. For metal–cage (M–cage) modes, in Section 2.4 we indicate the atomic displacements for both IR-active vibrations and vibrations that do not appear in the IR spectra. Some of these modes are illustrated by short movies in the Supplementary Materials. In particular, for some M–cage modes we explain the difference in IR activity between mono- and di-EMFs. The calculated spectra are discussed in detail in Section 2.5 for the mono-EMFs and in Section 2.6 for the di-EMFs. The comparison of the spectra calculated for different mono- and di-EMFs is given in Section 2.7. The full list of the calculated IR lines is given in the Appendix A; a visualization of the atom motion for some vibrational modes is presented in the Supplementary Materials (short movies).

## 2. Results and Discussion

### 2.1. Structure of M@C_60_ and M_2_@C_60_ Endofullerenes

According to the obtained results of quantum-chemical calculations, metal atoms are attached to the six-membered carbon ring, which is consistent with the data for the Y_2_@C_80_ endofullerene [53].

**Figure 1:**
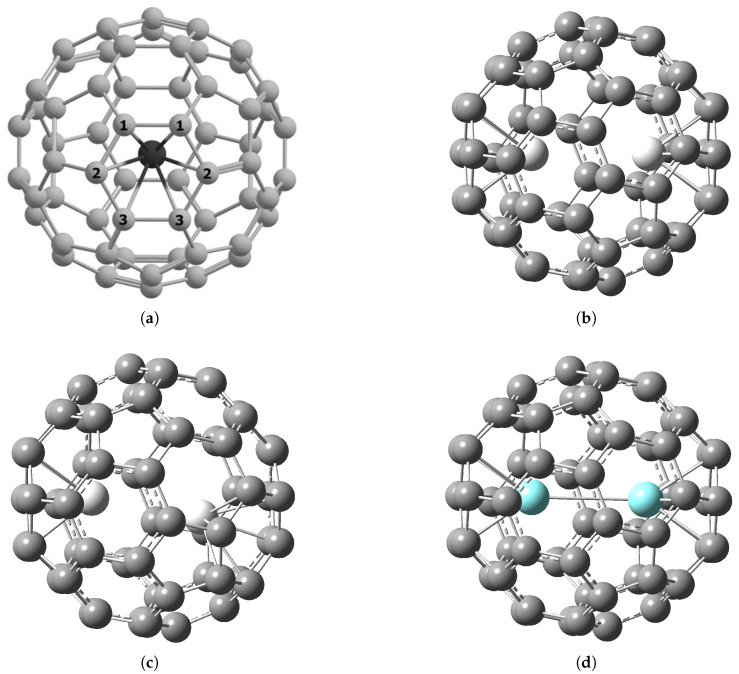
Structure of M_2_@C_60_ endofullerenes: (a) M@C_60_ structure, numeration of carbon atoms closest to the metal atom. (b) Sc_2_@C_60_, isomer of C_3v_ symmetry. (c) Sc_2_@C_60_, isomer of C_s_ symmetry. (d) Y_2_@C_60_, isomer of C_3v_ symmetry.

The calculated structures of endofullerenes are shown in Figure 1. The interatomic distances in the calculated structures are given in Table 1, where C_1_, C_2_, C_3_ are the atoms of the six-membered carbon ring to which the metal atom is attached (see Figure 1a). The distance between the metal atoms inside the Sc_2_@C_60_ ($C_{3v}$) fullerene is larger than for Y_2_@C_60_ ($C_{3v}$), because as the size of the metal atom increases, the metal–carbon bond length increases and the metal atoms shift closer to the center of the fullerene. For di-EMFs, the metal–carbon bond lengths are the same for both metal atoms. The growth of the metal–carbon bond length limits the maximum possible distance between metal atoms in the case of their attachment to opposite six-membered carbon rings. In the case of $C_{s}$ symmetry in Sc_2_@C_60_ the second metal atom is attached not to the opposite six-membered ring, but to the adjacent one, so the distance between the metal atoms is significantly smaller than in Sc_2_@C_60_ of $C_{3v}$ symmetry. Note that spin unrestricted DFT calculations show that the metal atom in Gd@C_60_ EMF situated opposite to the center of a six-membered ring bond [35].

The calculated metal–carbon bond lengths are in agreement with X-ray structural data for Sc_2_@C_82_ (bond lengths *r*(Sc–C) = 2.145–2.225 Å [54]) and Y_2_@C_79_N (bond lengths *r*(Y–C) = 2.336–2.523 Å [55]).

### 2.2. Energy Stability and Availability of M@C_60_ and M_2_@C_60_ Structures

The geometry optimization was performed with different initial arrangements of metal atoms. To calculate the spectroscopic parameters for each EMF, the structures with the lowest energy were selected. The following stable isomers of the considered EMFs have been found during the geometry optimization in our DFT calculations.

For the Sc_2_@C_60_ molecule, the lowest electronic state energy (i.e., zero relative energy) is achieved for the $C_{3v}$ symmetry isomer. The closest Sc_2_@C_60_ ground state (relative energy 0.11 eV) value is found for the isomer with the $C_{s}$ symmetry. For Y_2_@C_60_, the lowest ground electronic state (relative energy 0 eV) is achieved for the $C_{3v}$ isomer. Although the Y_2_@C_60_ isomer with the $C_{s}$ symmetry is close in energy (relative energy 0.20 eV), this isomer is unstable (i.e., has imaginary frequency *i*66.41 cm^−1^) and is not considered further. The imaginary frequency indicates that the structure corresponds not to a minimum, but to a saddle point on the potential energy surface. As for mono-EMFs (Sc@C_60_ and Y@C_60_), both of them are characterized by the $C_{s}$ symmetry of the lowest stable state. All structures described here are stable, as evidenced by the absence of imaginary frequencies of molecular vibrations.

The C_60_ fullerene features a relatively small inner cavity (∼0.7 nm in diameter), which imposes severe steric restrictions for encapsulation of atoms. While C_60_ can host up to three small Li atoms [56] or even LiF molecule [57], it is much harder to encapsulate metal atoms such as Sc (atomic radius ∼1.62 Å) and Y (atomic radius ∼1.80 Å). Larger metals like Y cause steric and strain effects that destabilize the cage–metal complex. At the same time, larger C_2n_ cages (e.g., with 2*n* = 80–84) can encapsulate two [58,59] or even three Sc atoms [60]. As for the C_60_ cage, DFT calculations [56] predict that, for instance, the production yield of Li_x_@C_60_ EMFs in the high-temperature synthesis should decrease by several orders of magnitude with *x* increasing from 1 to 3.

In addition to the structure stability, another explanation of difficulties with experimental synthesis of the considered EMFs can be high-temperature synthesis bias towards larger cages. The primary synthesis method for EMFs is arc-discharge or laser vaporization of metal/graphite composites. Under such high-temperature conditions, carbon cages that encapsulate metals typically favor larger cage sizes to ensure enough space in the inner cavity to place the metal atoms and achieve a local minimum of the potential energy surface of the EMF. Formation of pure C_60_ cages encapsulating Sc or Y atoms can thereby disfavored kinetically and thermodynamically.

It is possible to determine the molecular formula and structure only for those EMFs that can be extracted from arc discharge soot. Some EMFs can be synthesized but not detected if they are not soluble in commonly used solvents. The reason for poor solubility is low kinetic stability, which implies that the “insoluble” EMFs react with other carbon-containing substances formed in the soot, or polymerize. Then these EMFs can be extracted by modifying the synthesis protocol. Ensuring kinetic stability and solubility can be accomplished by electron transfer and/or chemical functionalization [50].

### 2.3. Vibrational Spectra of the M@C_60_ and M_2_@C_60_ Endofullerenes

The empty C_60_ fullerene has high $I_{h}$ symmetry, and theoretical calculations [61] show that of the 46 non-degenerate vibrational modes with different frequency, only four modes ($\nu_{1}=527$ cm^−1^, $\nu_{2}=576$ cm^−1^, $\nu_{3}=1182$ cm^−1^ and $\nu_{4}=1429$ cm^−1^ [62]) are IR-active and only ten modes are Raman, while the remaining “silent” modes appear in neutron scattering experiments.

The vibrational spectra of endohedral fullerene differ significantly from those of empty fullerene.

First, it occurs due to the involvement of metal atoms in the vibrations of the carbon cage. In particular, our calculation shows that the maximal contribution (10–30%) of the metal atoms takes place for the vibrational $\nu_{1}$–$\nu_{4}$ modes of M_2_@C_60_ di-EMFs and $\nu_{1}$–$\nu_{3}$ modes of M@C_60_ mono-EMFs. The frequency 102 cm^−1^ of shear vibrations of the Y_2_ dimer along the axis perpendicular to the Y–Y bond was calculated in Ref. [63] for Y_2_@C_84_ endofullerene. Raman measurements performed in the same study (Ref. [63]) for molecules in crystalline form gave crystal field splitting results of 77–95 cm^−1^ (T=100 K) for metal–cage vibrations. These results are in good agreement with our $\nu_{3,4}\approx$ 101–103 cm^−1^ values. The similar frequency calculated in Ref. [63] for Sc_2_@C_84_ is 104 cm^−1^, while the experimental Raman values lie between 84 and 110 cm^−1^ (T=100 K). Our calculated values $\nu_{1,4}\approx$ 79–94 cm^−1^ for Sc_2_@C_60_ ($C_{s}$) and $\nu_{1,2}\approx 82$ cm^−1^ for Sc_2_@C_60_ ($C_{3v}$) demonstrate overall agreement with the results of Ref. [63].

Second, the symmetry reduction due to the encapsulation of the metal atom leads to the appearance of lines in the IR spectra of endohedral fullerenes in the of 20–1620 cm^−1^ range. Most of these lines are absent both in the IR and Raman spectra of individual empty C_60_ fullerene [61,62] molecules, but some of the vibrational modes can occur in the IR and Raman spectra of C_60_ crystals (e.g., because of symmetry reduction due to crystal field effects and the presence of different isotopologues [64]).

The vibrations of the carbon cage (in both endohedral and empty fullerenes) are divided into radial (most lines with ν<800 cm^−1^) and tangential (ν>1000 cm^−1^) modes. While tangential modes preserve the shape of the carbon cage, radial modes change its shape. The IR-active C_60_ modes with frequencies 527 and 576 cm^−1^ are associated with radial motion of carbon atoms, while the 1182 and 1429 cm^−1^ modes are associated with tangential motion of carbon atoms [65,66].

The so-called “pumpkin” modes are accompanied by a deformation of the carbon cage from a oblate spheroid to a prolate one (much like squeezing and releasing a pumpkin or rubber ball). This type of vibration changes the shape of the carbon cage, while preserving (to first order) its overall volume.

For empty C_60_, all the five “pumpkin” modes are IR-inactive; they appear in Raman spectra and have the same frequency of 272 cm^−1^ [67]. Unlike the case of empty fullerene, in endohedral fullerenes the degeneracy of these modes is removed. This type of vibration changes the size of the polarized endofullerene cage, which changes the dipole moment and makes the pumpkin vibrational modes of the mono-EMF carbon cage IR-active. The difference between the frequencies of the EMF pumpkin modes and the corresponding C_60_ frequencies is due to the formation of metal–carbon bonds.

**Figure 2:**
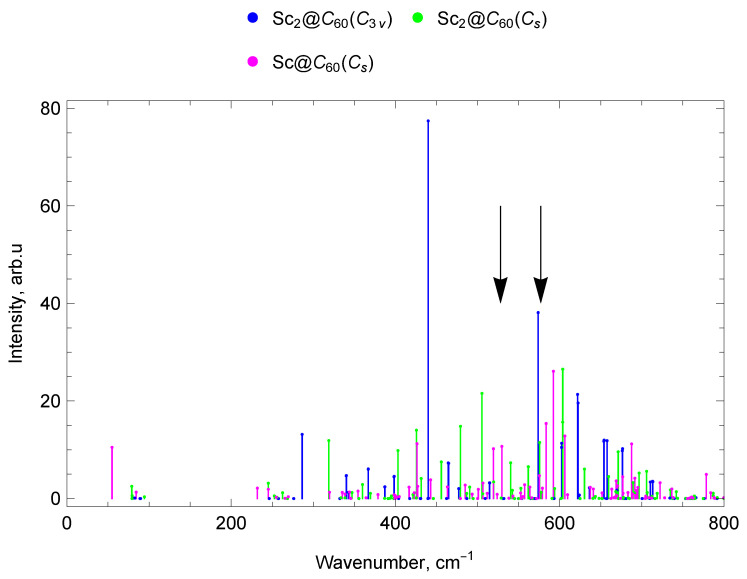
Comparison of IR spectra of scandium mono- and di-EMFs. The black arrows show the positions of two IR lines of the empty C_60_ fullerene ($\nu_{1}=527$ cm^−1^and $\nu_{2}=576$ cm^−1^ [62]).

**Figure 3:**
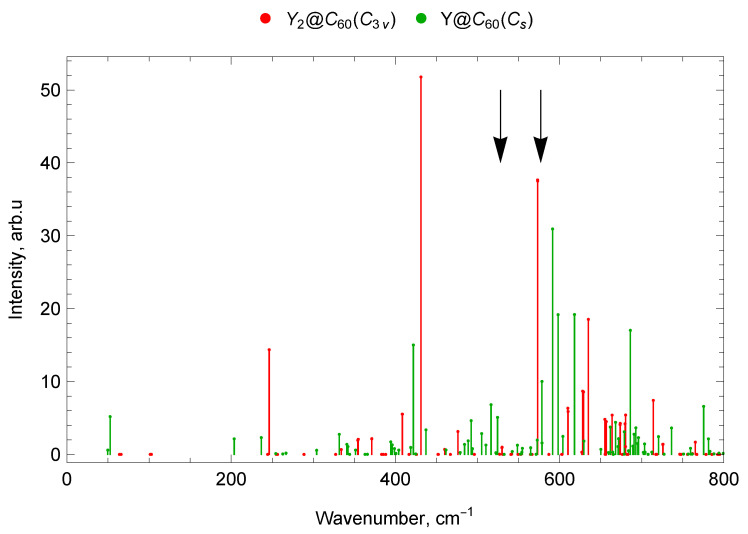
Comparison of IR spectra of yttrium mono- and di-EMFs. The black arrows show the positions of two IR lines of the empty C_60_ fullerene ($\nu_{1}=527$ cm^−1^and $\nu_{2}=576$ cm^−1^ [62]).

The “silent” mode with a frequency of 403 cm^−1^ does not appear in either IR or Raman spectra, but it is registered in neutron scattering experiments [67,68]. This vibrational mode is the only tangential mode with a frequency significantly lower than 1000 cm^−1^. Unlike all other tangential modes, large fragments of the carbon cage are involved in the coordinated motion in this mode, which reduces the vibration frequency. This mode represents counter torsional vibrations of two carbon cage’s hemispheres. Due to the decrease in symmetry upon encapsulation of the metal dimer, this mode occurs IR-active for Sc_2_@C_60_ ($C_{3v}$), having a frequency of $\nu_{18}=387.15$ cm^−1^ and a significant intensity (see Figure 2). For the Sc_2_@C_60_ ($C_{s}$) isomer, this mode is also IR-active, with a frequency of $\nu_{20}=395.12$ cm^−1^, but low intensity (see Figure 2). This mode is also IR-active for Y_2_@C_60_ ($C_{3v}$), with a frequency of $\nu_{16}=371.3$ cm^−1^ and significant intensity (see Figure 3). For Sc@C_60_ and Y@C_60_, this type of vibrations gives several lines of medium and low intensity in the IR spectrum in the range of 394–406 cm^−1^. For Sc@C_60_ this mode has a frequency of $\nu_{16}=398.32$ cm^−1^, but a weak intensity (see Figure 2). For Y@C_60_ this mode appears in the IR spectrum at a frequency of $\nu_{16}=394.37$ cm^−1^ (see Figure 3). Activation of the “silent” mode of C_60_ is observed due to the influence of the encapsulated metal atom or metal dimer.

The “Breathing” mode represents vibrations with all-round compression–extension of the fullerene’s carbon cage. This mode for empty C_60_ has a frequency of 496 cm^−1^, this mode is not IR-active and manifests itself in the Raman spectra [62]. Sc@C_60_ and Y@C_60_ vibrations with close to isotropic compression–extension of the carbon framework have a frequency of 484–485 cm^−1^; due to the reduction of symmetry, these vibrations are IR-active, but the corresponding spectral lines are weak. For Sc_2_@C_60_ ($C_{3v}$) and Y_2_@C_60_ ($C_{3v}$), these vibrations are symmetrical with respect to the fullerene center and therefore are not IR-active. However, for Sc_2_@C_60_ ($C_{s}$), due to the reduced symmetry the “breathing” mode appears in the IR spectrum with a frequency $\nu_{28}=455.84$ cm^−1^ and a significant intensity (see Figure 2). The encapsulation of scandium dimer leads to the appearance of IR activity in this Raman mode of C_60_ fullerene.

Of the tangential vibrations, the pentagonal pinch mode should be mentioned. It is caused by the absolutely symmetric stretching of the pentagonal rings of the carbon cage. The corresponding vibrations are not IR-active for all structures considered here, and their frequencies lie in the range 1502–1512 cm^−1^ (see Table A1 and Table A2). These values are in order of magnitude consistent with the frequency of the 1470 cm^−1^ pentagonal-pinch Raman active mode of the empty C_60_ fullerene, which corresponds to the displacement of carbon atoms to the 12 centers of the pentagonal rings along the fullerene surface [67].

**Figure 4:**
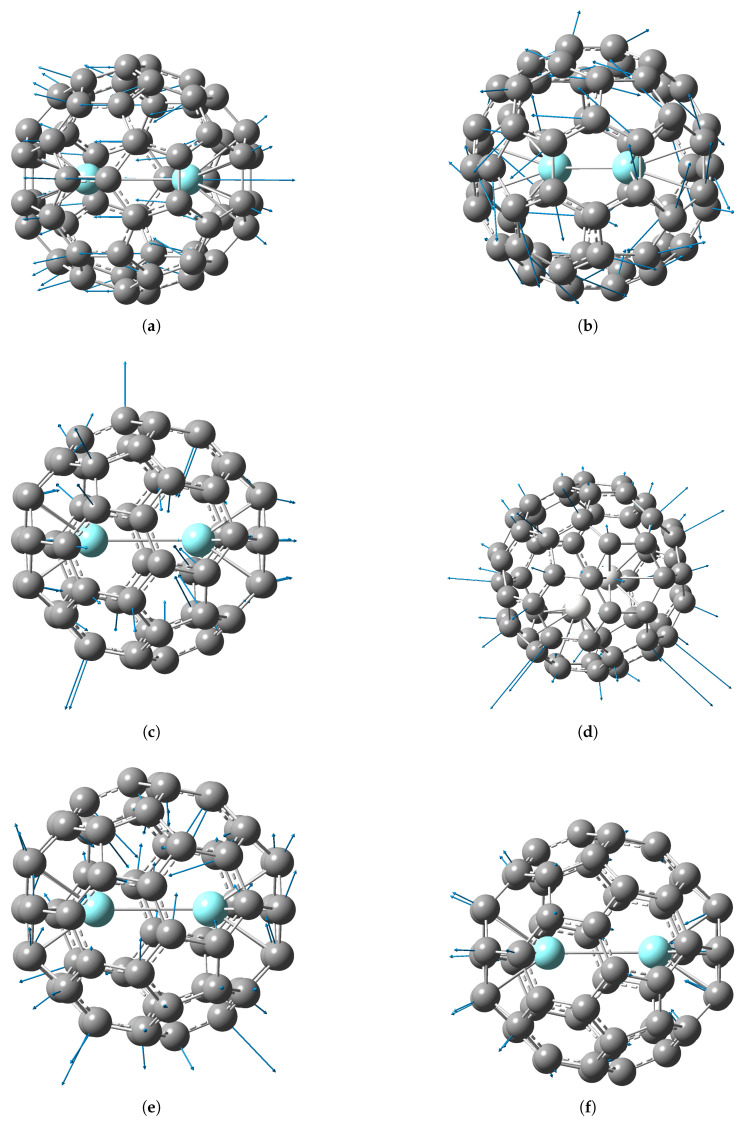
IR-active vibrational modes in M_2_@C_60_ di-EMFs (the arrows show the directions of atomic displacements): (a) $\nu_{6}$ M–cage mode in $Y_{2}@C_{60}\;\left(C_{3v}\right)$. (b) $\nu_{16}$ “silent” mode in $Y_{2}@C_{60}\;\left(C_{3v}\right)$ (counter-torsional vibrations of the cage hemispheres). (c) $\nu_{26}$ radial mode in $Y_{2}@C_{60}\;\left(C_{3v}\right)$ (strong line in IR spectrum). (d) $\nu_{28}$ “breathing” radial mode in ${\mathrm{Sc}}_{2}@C_{60}\;\left(C_{s}\right)$. (e) $\nu_{48}$ radial mode in $Y_{2}@C_{60}\;\left(C_{3v}\right)$ (strong line in IR spectrum). (f) $\nu_{57}$ radial mode in $Y_{2}@C_{60}\;\left(C_{3v}\right)$ (strong line in IR spectrum). A visualization of the atom motion for some vibrational modes is presented in the Supplementary Materials (short movies).

**Figure 5:**
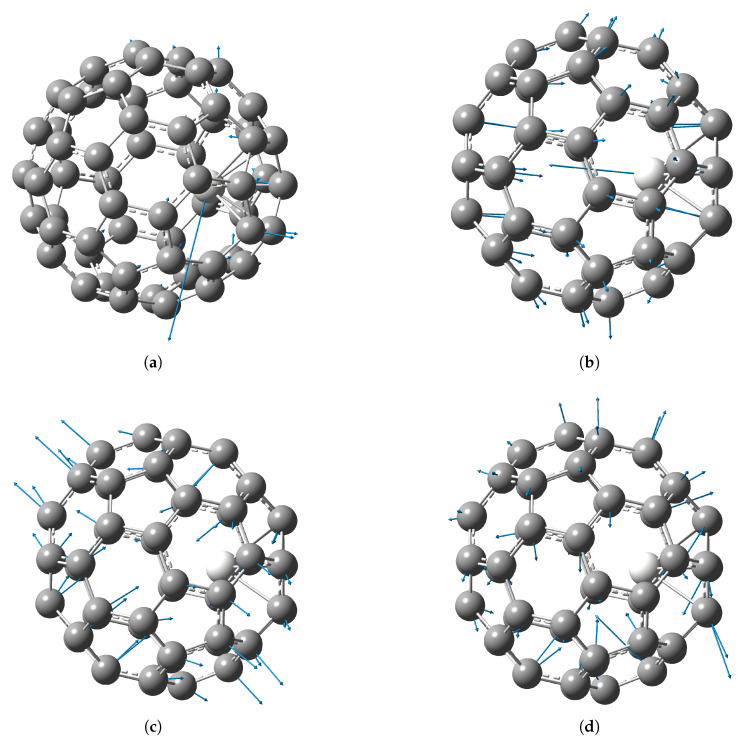
IR-active (unless otherwise specified) vibrational modes in M@C_60_ mono-EMFs (the arrows show the directions of atomic displacements): (a) $\nu_{1}$ M–cage mode in $\mathrm{Sc}@C_{60}\;\left(C_{s}\right)$. (b) $\nu_{3}$ M–cage “pumpkin” mode in $\mathrm{Sc}@C_{60}\;\left(C_{s}\right)$. (c) $\nu_{6}$ “pumpkin” (IR-inactive) mode in $\mathrm{Sc}@C_{60}\;\left(C_{s}\right)$. (d) $\nu_{48}$ radial mode in $\mathrm{Sc}@C_{60}\;\left(C_{s}\right)$ (strong line in IR spectrum). A visualization of the atom motion for some vibrational modes is presented in the Supplementary Materials (short movies).

The directions of atomic displacement most typical for the structures calculated in this work are shown by arrows in Figure 4 and Figure 5. Additionally, a visualization of the atom motion for some vibrational modes is presented in the Supplementary Materials (short movies).

### 2.4. M–Cage Modes in Mono- and di-EMFs

Compared to empty C_60_ fullerene, the IR spectrum of both mono- and di-EMFs contains significantly more lines. The spectra of di-EMFs with $C_{3v}$ symmetry in the ranges 20–-450, 500–800, and 1200–1600 cm^−1^ have fewer IR-active vibrational modes compared to mono-EMFs. Indeed, according to our calculations of mono-EMFs, the metal atom is not located in the center of the M@C_60_ molecule, so it cannot have central symmetry. Geometry optimization lead to different EMF isomers with $C_{3v}$ and $C_{s}$ symmetry. In M_2_@C_60_ with $C_{3v}$ symmetry, both the metal atoms are located on the $C_{3}$ axis at the same distance from the center of C60 and are bonded to opposite six-membered rings.

Therefore, the di-EMF M_2_@C_60_ molecules of $C_{3v}$ have a center of symmetry. This higher (as compared to M_2_@C_60_) symmetry prohibits some IR lines. For Sc_2_@C_60_ of $C_{s}$ symmetry, this effect is less pronounced due to the metal atoms bonding with the six-membered rings that are placed near the $C_{3}$ axis (but not exactly on it).

The symmetry considerations explain why a number of M–cage modes in di-EMFs are not IR-active. Our calculations show that the charge density is positive at the incapsulated metal atom locations. In mono-EMF M@C_60_, therefore, a motion of the metal atom (which always takes place in M–cage modes) results in a change of the positive charge center. In turn, this displacement of positive charge leads to change of the molecule dipole moment that makes the corresponding vibration mode IR-active. On the contrary, in di-EMFs M_2_@$C_{60}$, the metal ions move in opposite directions (with respect to the center of symmetry) in all vibrational modes except for shear vibrations. Therefore most of M–cage modes vibrations are IR-inactive for di-EMFs.

Thus, in the IR spectrum of Y_2_@C_60_ compared to Y@C_60_, the intensity of all M-cage modes (in the 20–450 cm^−1^ range) decreases sharply, with the exception of $\nu_{6}$ and $\nu_{26}$, associated with shear vibrations of the metal dimer along the Y–Y bond. This effect is caused by the symmetrical motion of metal atoms relative to the center of the fullerene, while the center of positive charge does not shift and the vibrations do not change the dipole moment of the molecule and therefore are not IR-active.

Similarly, in the IR spectrum of Sc_2_@C_60_ ($C_{3v}$) compared to Sc@C_60_ (in the 20–450 cm^−1^ range), with the exception of modes associated with shear vibrations of the metal dimer along the Sc–Sc bond. For Sc_2_@C_60_ ($C_{s}$), this effect also occurs, but is less pronounced.

### 2.5. Spectra of Mono-EMFs of Sc and Y

**Figure 6:**
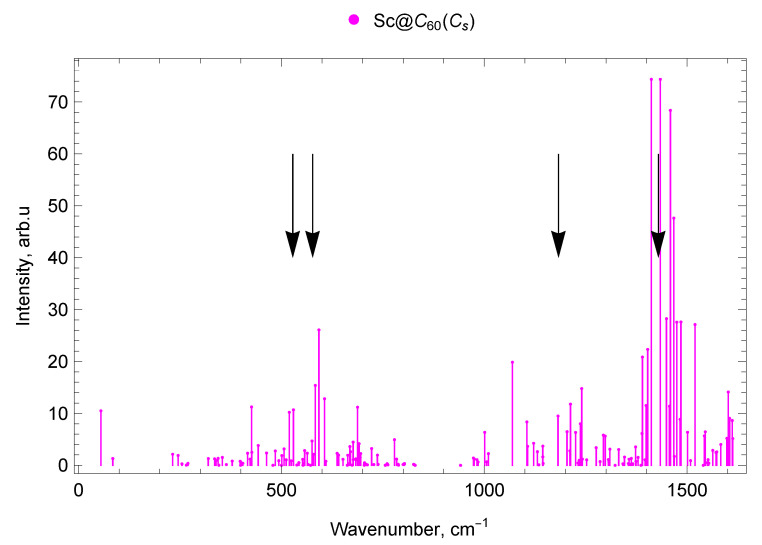
IR spectrum of Sc@C_60_ endofullerene (isomer of $C_{s}$ symmetry). The black arrows show the positions of four IR lines of the empty C_60_ fullerene ($\nu_{1}=527$ cm^−1^, $\nu_{2}=576$ cm^−1^, $\nu_{3}=1182$ cm^−1^ and $\nu_{4}=1429$ cm^−1^ [62]).

**Figure 7:**
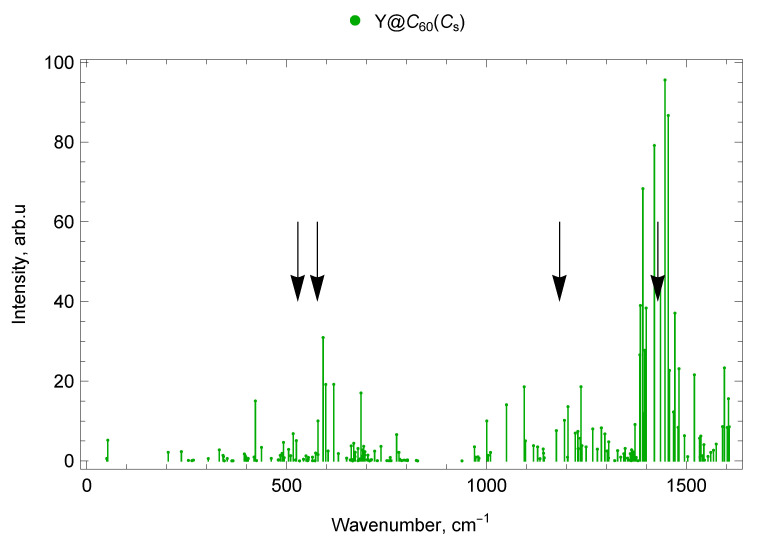
IR spectrum of Y@C_60_ endofullerene (isomer of $C_{s}$ symmetry). The black arrows show the positions of four IR lines of the empty C_60_ fullerene ($\nu_{1}=527$ cm^−1^, $\nu_{2}=576$ cm^−1^, $\nu_{3}=1182$ cm^−1^ and $\nu_{4}=1429$ cm^−1^ [62]).

The calculated vibrational IR spectra of mono-EMFs are presented in Figure 6 and Figure 7. The black arrows show the positions of four IR lines of the empty C_60_ fullerene ($\nu_{1}=527$ cm^−1^, $\nu_{2}=576$ cm^−1^, $\nu_{3}=1182$ cm^−1^ and $\nu_{4}=1429$ cm^−1^ [62]).

#### 2.5.1. Sc@C_60_ ($C_{s}$)

In the Sc@C_60_ spectrum, there are seven vibrational modes in the frequency range up to 300 cm^−1^: one IR-active mode (with a frequency of 55 cm^−1^), three weak (low-intensity) IR lines, and three IR-inactive modes. Between 300 and 400 cm^−1^, there are 11 modes, of which 8 are low-intensity IR and 3 are IR-inactive. The range 400–600 cm^−1^contains 6 IR-active lines (with frequencies 426, 519, 530, 575, 583, 592 cm^−1^), 16 weak IR lines, and 9 IR-inactive modes (31 modes in total). There are 40 vibrational modes in the 600–800 cm^−1^ range, of which 4 are IR-active (606, 677, 688, 779 cm^−1^), 17 weak IR lines, and 19 IR-inactive modes (see Figure 2).

In the 800–1000 cm^−1^ spectral range, 11 vibrational Sc@C_60_ modes were found, of which 4 are low-intensity IR and 7 are IR-inactive. In the range 1000–1200 cm^−1^, there are 13 vibrational modes, of which 4 are IR-active (with frequencies 1001, 1069, 1104, 1181 cm^−1^), 7 are IR-weak, and 2 are IR-inactive. In addition, 35 vibrational modes fall within the frequency range 1200–1400 cm^−1^; among them, 10 are IR-active (1204, 1212, 1225, 1237, 1240, 1293, 1298, 1389, 1390, 1399 cm^−1^), 18 low-intensity IR lines, and 7 IR-inactive modes. In the range 1400–1600 cm^−1^ 19 IR-active lines were found (with frequencies 1403, 1412, 1434, 1449, 1456, 1459, 1467, 1474, 1483, 1485, 1501, 1519, 1542, 1545, 1598, 1601, 1605, 1611, and 1612 cm^−1^), 8 low-intensity IR lines, and 2 IR-inactive modes (29 modes in total; see Figure 6).

#### 2.5.2. Y@C_60_ ($C_{s}$)

In the Y@C_60_ spectrum, there are seven vibrational modes in the frequency range up to 300 cm^−1^: one IR-active (with a frequency of 53 cm^−1^), three low-intensity IR, and three IR-inactive. Between 300 and 400 cm^−1^, 12 modes were found, 8 of which are low-intensity IR and 4 are IR-inactive. The range 400–600 cm^−1^ contains 6 IR-active lines (with frequencies 422, 516, 524, 578, 591, 598 cm^−1^), 15 low-intensity IR lines, and 10 IR-inactive modes (31 modes in total). There are 40 vibrational modes in the 600–800 cm^−1^ range; of which 4 are IR-active (618, 668, 686, 775 cm^−1^), 17 are low-intensity IR, and 19 are IR-inactive (see Figure 7).

In the range 800–1000 cm^−1^, there are 10 Y@C_60_ modes: 4 low-intensity IR modes and 6 IR-inactive modes. Between 1000 and 1200 cm^−1^, there are 14 vibrational modes, of which 6 are IR-active (with frequencies 1001, 1050, 1095, 1098, 1175, 1195 cm^−1^) and 8 are low-intensity IR lines. Additionally, 36 vibrational modes fall within the frequency range 1200–1400 cm^−1^; of these, 14 are IR-active (1204, 1223, 1229, 1235, 1237, 1266, 1288, 1297, 1372, 1384, 1385, 1392, 1396, 1400 cm^−1^), 19 are low-intensity IR, and 3 are non-IR-active. In the range 1400–1600 cm^−1^ we found 18 IR-active lines (with frequencies 1420, 1436, 1447, 1455, 1458, 1468, 1472, 1480, 1482, 1496, 1521, 1534, 1537, 1591, 1596, 1603, 1606, and 1608 cm^−1^), 8 low-intensity IR lines, and 1 IR-inactive mode (27 modes in total; see Figure 3).

### 2.6. Spectra of di-EMFs of Sc and Y

**Figure 8:**
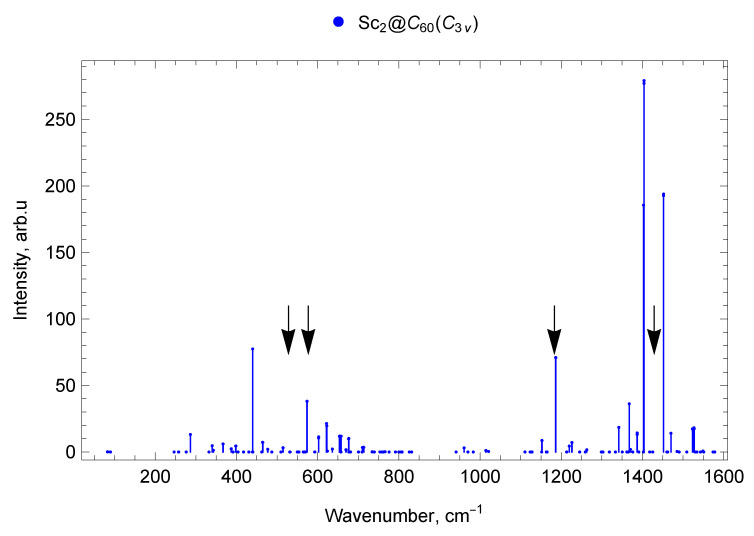
Vibrational spectrum of Sc_2_@C_60_ endofullerene (isomer of $C_{3v}$ symmetry). The black arrows show the positions of four IR lines of the empty C_60_ fullerene ($\nu_{1}=527$ cm^−1^, $\nu_{2}=576$ cm^−1^, $\nu_{3}=1182$ cm^−1^ and $\nu_{4}=1429$ cm^−1^ [62]).

**Figure 9:**
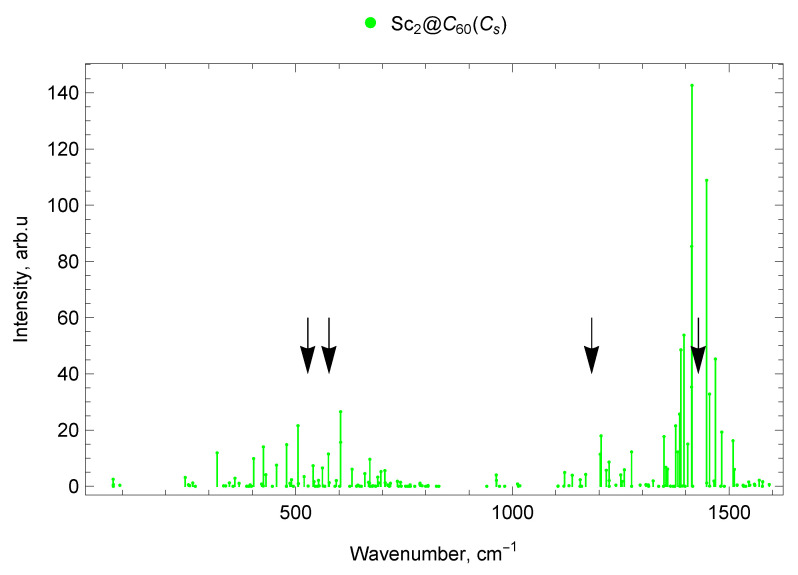
Vibrational spectrum of Sc_2_@C_60_ endofullerene (isomer of $C_{s}$ symmetry). The black arrows show the positions of four IR lines of the empty C_60_ fullerene ($\nu_{1}=527$ cm^−1^, $\nu_{2}=576$ cm^−1^, $\nu_{3}=1182$ cm^−1^ and $\nu_{4}=1429$ cm^−1^ [62]).

**Figure 10:**
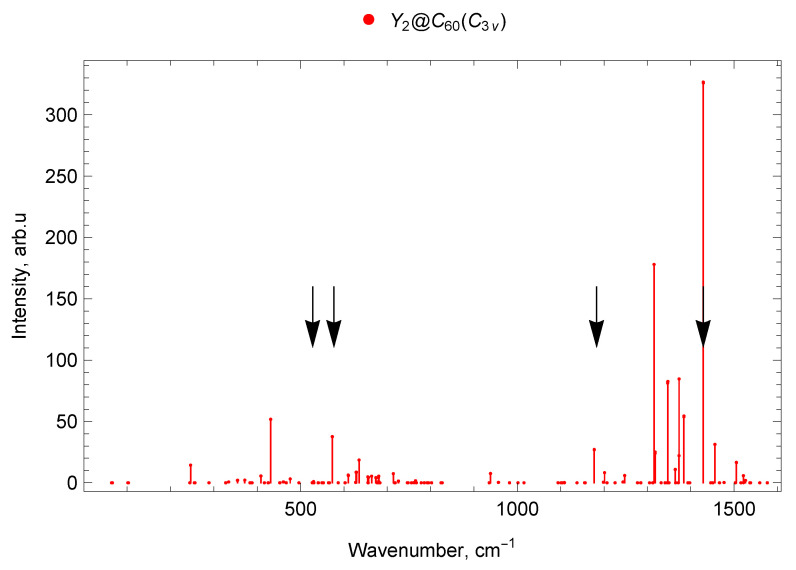
Vibrational spectrum of Y_2_@C_60_ endofullerene (isomer of $C_{3v}$ symmetry). The black arrows show the positions of four IR lines of the empty C_60_ fullerene ($\nu_{1}=527$ cm^−1^, $\nu_{2}=576$ cm^−1^, $\nu_{3}=1182$ cm^−1^ and $\nu_{4}=1429$ cm^−1^ [62]).

The calculated vibrational IR spectra of di-EMFs are presented in Figure 8, Figure 9 and Figure 10. The black arrows show the positions of four IR lines of the empty C_60_ fullerene ($\nu_{1}=527$ cm^−1^, $\nu_{2}=576$ cm^−1^, $\nu_{3}=1182$ cm^−1^ and $\nu_{4}=1429$ cm^−1^ [62]).

#### 2.6.1. Sc_2_@C_60_ ($C_{3v}$)

The vibrational spectrum of Sc_2_@C_60_ with $C_{3v}$ symmetry is presented in Figure 8. The vibrational modes $\nu_{1}$ = 81.95 cm^−1^ and $\nu_{2}$ = 82.68 cm^−1^ represent shear vibrations of the Sc_2_ dimer along the axis perpendicular to the Sc–Sc bond, the fraction of metal atom involvement being 17%. The vibrational modes $\nu_{3}$ = 89.02 cm^−1^ and $\nu_{4}$ = 89.65 cm^−1^ correspond to torsional vibrations of the Sc_2_ dimer with respect to the axis passing perpendicularly through the middle of the metal–metal bond (lateral M–cage mode); the fraction of metal atoms involved is 22%. This type of vibrations is not IR-active, since it does not lead to the displacement of the center of positive charge.

Of the pumpkin modes, the largest fraction (4%) of the involvement of Sc atoms is characteristic of the mode $\nu_{5}$ = 246.35 cm^−1^ (compression–stretching of the Sc_2_ dimer along the Sc–Sc bond). In pumpkin modes $\nu_{6}$–$\nu_{7}$ with frequencies 257–258 cm^−1^ the compression–stretching of the carbon cage goes perpendicular to the Sc–Sc bond. Metal atoms are almost not involved; these vibrations do not appear in IR spectra, as well as in the empty C_60_ fullerene spectrum. In pumpkin modes $\nu_{8}$–$\nu_{9}$ with frequencies ≈276 cm^−1^ the carbon cage oscillates with partial involvement of scandium atoms, the metal atoms oscillate symmetrically with respect to the center of the fullerene. These modes are IR inactive.

In contrast to Sc_2_@C_60_ (C_3v_), the pumpkin modes of Sc@C_60_ IR are active because of the involvement of the metal atom and the change in the position of the positive charge center during oscillation. The frequencies of the pumpkin modes of scandium mono-EMF are 231.91, 245.22, 254.84, 265.83, 269.56 cm^−1^, which are close to those of the di-EMF.

The vibrational mode $\nu_{10}$ = 286.41 cm^−1^ is IR-active (see Figure 8) and represents shear vibrations of the Sc_2_ dimer along the Sc–Sc bond, with 7% involvement of metal atoms.

The vibrational mode $\nu_{23}$ = 417.35 cm^−1^ is a combination of compression–stretching of the Sc_2_ dimer and vibrations of the carbon cage atoms with 4% of the metal atoms involved. This mode does not appear in the IR spectrum, since the vibrations of metal atoms are symmetric with respect to the fullerene center and therefore do not change the dipole moment of the molecule.

The vibrational mode $\nu_{27}$ = 439.88 cm^−1^ is IR-active (see Figure 8); it is a combination of shear vibrations of the Sc_2_ dimer along the Sc–Sc bond and pentagon radial modes of the fullerene, with 1% involvement of metal atoms.

The vibrations with symmetric compression of the pentagonal rings of the carbon cage (pentagonal-pinch mode) have frequency $\nu_{167}=1509.15$ cm^−1^ and are not IR-active. In an empty fullerene, this vibrational mode appears in the Raman spectrum and has a frequency of 1470 cm^−1^ [67].

In the spectrum of Sc_2_@C_60_ (C_3v_) in the frequency range up to 300 cm^−1^ there are 10 vibrational modes, of which 1 is IR-active (with a frequency of 286 cm^−1^) and 9 are IR-inactive. In the range from 300 to 400 cm^−1^, there are 11 modes, which correspond to 6 IR-active modes (with frequencies 340 (doublet of close lines), 367 (doublet), 398, 399 cm^−1^), 3 weak/low-intensity IR lines, and 2 IR-inactive modes. In the range 400–600 cm^−1^ there are 5 IR-active lines (with frequencies 440, 464, 465, 574, 574 cm^−1^), 3 weak IR lines, and 22 IR-inactive modes (30 modes in total). Of the 40 vibrational modes in the range 600–800 cm^−1^, there are 9 IR-active modes (602, 602, 622 (doublet), 654 (doublet), 657, 676, 677 cm^−1^), 7 weak IR lines, and 24 IR-inactive modes (see Figure 2).

In the spectral range 800–1000 cm^−1^, there are 12 Sc_2_@C_60_ (C_3v_) vibrational modes, of which 1 is weak IR mode and 11 are IR-inactive. In the range 1000–1200 cm^−1^, there are 16 vibrational modes, of which 4 are IR-active (with frequencies 1152, 1153, 1186 (doublet) cm^−1^), 2 are low-intensity IR lines, and 10 are IR-inactive. Of the 30 vibrational modes in the range 1200–1400 cm^−1^, there are 8 IR-active modes (1220 (doublet), 1226, 1342 (doublet), 1367, 1387 (doublet) cm^−1^), 3 low-intensity IR lines, and 19 IR-inactive modes. In the 1400–1600 cm^−1^ range, there are 10 IR-active lines (with the frequencies 1403, 1404 (doublet), 1452 (doublet), 1470, 1523 (doublet), 1527 (doublet) cm^−1^), 1 low-intensity IR line, and 20 IR-inactive modes (31 modes in total; see Figure 8).

#### 2.6.2. Sc_2_@C_60_ ($C_{s}$)

The vibrational spectrum of Sc_2_@C_60_ with $C_{s}$ symmetry is presented in Figure 9.

The vibrational modes $\nu_{1}$ = 78.95 cm^−1^ and $\nu_{4}$ = 94.37 cm^−1^ represent shear vibrations of the Sc_2_ dimer along the axis perpendicular to the Sc–Sc bond, the fraction of metal atoms involvement being 10–17%. The vibrational modes are IR-active, which is due to the asymmetric position of metal atoms relative to the center of the fullerene.

The vibrational modes $\nu_{2}$ = 79.24 cm^−1^ and $\nu_{3}$ = 79.39 cm^−1^ are torsional vibrations of the Sc_2_ dimer around an axis passing perpendicularly through the middle of the metal–metal bond (lateral M–cage mode), the fraction of metal atoms involvement being 22–29%.

For Sc_2_@C_60_ ($C_{s}$), the pumpkin modes $\nu_{5}$–$\nu_{9}$ have frequencies of 245.22 cm^−1^, 252.59 cm^−1^, 255.78 cm^−1^, 262.64 cm^−1^, and 268.56 cm^−1^. Of these vibrations, the highest IR activity has the $\nu_{5}$ mode, for which the compression–stretching of the carbon cage is combined with shear vibrations of the Sc_2_ dimer along the Sc–Sc bond, the fraction of metal atoms involvement being 3%.

The vibrational mode $\nu_{10}$ = 318.83 cm^−1^ is IR-active (see Figure 9) and represents shear vibrations of the Sc_2_ dimer along the Sc–Sc bond combined with deformations of the carbon cage, the fraction of metal atoms involvement being 2%.

The vibrational mode $\nu_{13}$ = 347.14 cm^−1^ is a combination of compression–stretching of the Sc_2_ dimer and vibrations of the carbon cage atoms, with 2% involvement of metal atoms.

The vibrations at frequency $\nu_{25}$ = 425.52 cm^−1^ are IR-active (see Figure 9). They are a combination of shear vibrations of the Sc_2_ dimer along the Sc–Sc bond and carbon cage radial mode, with 2% involvement of metal atoms.

In the spectrum of Sc_2_@C_60_ ($C_{s}$), there are nine vibrational modes in the frequency range up to 300 cm^−1^ of which four are low-intensity IR modes and five are IR-inactive modes. In the range from 300 to 400 cm^−1^, there are 12 modes: 1 IR-active (319 cm^−1^), 4 low-intensity IR, and 7 IR-inactive. In the range 400–600 cm^−1^, there are 8 IR-active lines (with frequencies 403, 426, 456, 479, 505, 540, 562, 576 cm^−1^), 11 low-intensity IR lines, and 10 IR-inactive modes (29 modes in total). Additionally, 42 vibrational modes fall within the frequency range 600–800 cm^−1^, of which 7 are IR-active (604 (doublet), 630, 660, 671, 697, 706 cm^−1^), 12 low-intensity IR modes, and 23 IR-inactive modes (see Figure 2).

In the spectral range up to 800–1000 cm^−1^, there are 11 modes of Sc_2_@C_60_ ($C_{s}$): 2 low-intensity IR and 9 IR-inactive modes. In the range 1000–1200 cm^−1^, there are 14 vibrational modes, of which 1 is IR-active (with a frequency of 1121 cm^−1^), 4 are low-intensity IR, and 9 are IR-inactive.

In the 1200–1400 cm^−1^ range, 34 vibrational modes were found: 14 IR-active (1203, 1205, 1217, 1223, 1258, 1275, 1350, 1354, 1358, 1377, 1382, 1386, 1389, 1396 cm^−1^), 9 low-intensity IR, and 11 IR-inactive.

Of the 29 modes falling within the range 1400–1600 cm^−1^, 10 lines are IR-active (with frequencies 1405, 1413, 1414 (doublet), 1448, 1455, 1468, 1483, 1509, 1512 cm^−1^), 9 low-intensity IR lines, and 10 IR-inactive modes (see Figure 9).

#### 2.6.3. Y_2_@C_60_ ($C_{3v}$)

The vibrational spectrum of Y_2_@C_60_ with $C_{3v}$ symmetry is presented in Figure 10.

The vibrational modes $\nu_{1}$ = 63.95 cm^−1^ and $\nu_{2}$ = 65.99 cm^−1^ represent torsional vibrations of the Y_2_ dimer around the axis perpendicular to the Y–Y bond, the fraction of metal atoms involvement being 25%. This type of vibrations does not displace the positive charge center and does not change the dipole moment of the molecule, so it does not appear in IR spectra.

The vibrational modes $\nu_{3}$ = 101.49 cm^−1^ and $\nu_{4}$ = 102.91 cm^−1^ represent shear vibrations of the dimer Y_2_ along the axis passing perpendicularly through the middle of the Y–Y bond, the fraction of metal atoms involvement being 15%.

For the Y_2_@C_60_ di-EMF, yttrium atoms are involved only in the pumpkin mode $\nu_{5}$ = 244.43 cm^−1^ (compression-extension of the Y_2_ dimer along the Y–Y bond), the fraction of metal atoms involvement is 5%. This mode is IR-inactive because the metal atoms oscillate symmetrically with respect to the fullerene center.

The vibrational mode $\nu_{6}$ = 246.34 cm^−1^ is IR-active (see Figure 10) and represents shear vibrations of the Y_2_ dimer along the Y–Y bond (longitudinal M–cage mode, antisymmetric motion of metal atoms with respect to the fullerene center), with 10% involvement of metal atoms.

In pumpkin modes $\nu_{7}$–$\nu_{8}$ with frequencies 255–256 cm^−1^ the compression-extension of the carbon cage is perpendicular to the Y–Y bond, metal atoms are practically not involved, and these vibrations do not appear in the IR spectra, as well as in the empty fullerene C_60_. In pumpkin modes $\nu_{9}$–$\nu_{10}$ with frequencies ≈289 cm^−1^ vibrations of the carbon cage go with partial involvement of yttrium atoms, metal atoms oscillate symmetrically relative to the center of the fullerene. These modes are IR inactive.

In contrast to the corresponding Y_2_@C_60_ modes, the pumpkin modes of Y@C_60_ are IR-active. The non-symmetric involvement of the metal atom in the vibrations leads to a change in the dipole moment of the Y@C_60_ molecule, and therefore to appearance of the corresponding lines in the IR spectrum. The frequencies of the pumpkin modes of yttrium mono-EMF are 203.71, 236.67, 254.30, 262.87, 266.81 cm^−1^; these lines, except for the first one, are close in frequency to the di-EMF lines.

The vibrational mode $\nu_{19}$ = 385.23 cm^−1^ is a combination of compression–stretching of the Y_2_ dimer and vibrations of the carbon cage atoms, the fraction of metal atoms involvement being 3%. This mode is not IR-active because the motion of metal atoms is symmetric with respect to the fullerene center.

The vibrational mode $\nu_{26}$ = 431.05 cm^−1^ is IR-active (see Figure 10). It is a combination of shear vibrations of the Y_2_ dimer along the Y–Y bond and oscillating vibrations of the pentagonal rings of the fullerene, with 1% involvement of metal atoms.

The vibrations with symmetric compression of the pentagonal rings of the carbon cage (pentagonal-pinch mode) have frequency $\nu_{167}=1502.95$ cm^−1^ and are not IR-active.

In the spectrum Y_2_@C_60_ ($C_{3v}$) in the frequency range up to 300 cm^−1^ there are ten vibrational modes, of which one is IR-active (with a frequency of 246 cm^−1^) and nine are IR-inactive. From 300 to 400 cm^−1^ there are ten modes: five IR low-intensity lines and five IR-inactive modes. The range 400–600 cm^−1^ contains 5 IR-active lines (with frequencies 408, 409, 431, 573 (doublet) cm^−1^), 5 low-intensity IR lines, and 19 IR-inactive modes (29 modes in total). Forty-four oscillatory modes were found in the range 600–800 cm^−1^: 10 IR-active (610 (doublet), 628, 629, 635, 655, 657, 664, 680, 714 cm^−1^), 6 low-intensity IR lines, and 28 IR-inactive modes. (see Figure 3).

In the spectral range 800–1000 cm^−1^ there are ten Y_2_@C_60_ ($C_{3v}$) modes, of which one is IR-active (938 cm^−1^) and nine are IR-inactive. In the range 1000–1200 cm^−1^, there are 18 vibrational modes, of which 2 are IR-active (with frequencies 1177, 1178 cm^−1^), 1 is weak IR line, and 15 modes are IR-inactive. Additionally, 35 vibrational modes fall within the frequency range 1200–1400 cm^−1^; of these, 13 are IR-active (1201, 1248, 1315, 1318, 1318, 1347 (doublet), 1364 (doublet), 1373 (doublet), 1384 (doublet) cm^−1^), 2 low-intensity IR lines and 20 modes are IR-inactive. In the 1400–1600 cm^−1^ range there are 7 IR-active lines (with frequencies 1429 (doublet), 1456, 1505, 1506, 1521, 1522 cm^−1^), 1 low-intensity IR line, and 16 IR-inactive modes (24 modes in total; see Figure 10).

### 2.7. Comparison of the Calculated Spectra

The spectra of mono- and di-EMFs of scandium are given in Figure 2 for comparison, the similar comparison for yttrium is given in Figure 3. The black arrows show the positions of two IR lines of the empty C_60_ fullerene ($\nu_{1}=527$ cm^−1^ and $\nu_{2}=576$ cm^−1^ [62]).

#### 2.7.1. Spectra of Scandium Mono- and di-EMFs

In the range up to 300 cm^−1^ for mono-EMF scandium Sc@C_60_, one intense line at 55 cm^−1^ (lateral M-cage mode) is observed, while for di-EMF Sc_2_@C_60_ ($C_{3v}$) there is also only one intense line at 286 cm^−1^ (pumpkin mode, which also includes the longitudinal M–cage mode). There are no intense IR lines of Sc_2_@C_60_ ($C_{s}$) di-EMF in this spectral range. In the frequency range 300–400 cm^−1^ Sc@C_60_ has no intense lines. However, for Sc_2_@C_60_ ($C_{3v}$), there is a group of lines with a frequency of 340–400 cm^−1^, and for Sc_2_@C_60_ ($C_{s}$) an intense line with a frequency of 319 cm^−1^is also observed. In the 400–800 cm^−1^ domain, IR-active lines with close frequencies are observed for both mono-EMF and both isomers of di-EMF scandium.

In the 800–1000 cm^−1^ range, there are no IR-active lines of noticeable amplitude for scandium EMFs, Sc@C_60_, Sc_2_@C_60_ ($C_{3v}$), Sc_2_@C_60_ ($C_{s}$). In the range 1000–1110 cm^−1^ there are IR-active modes of the mono-EMF and no IR-active lines of noticeable amplitude for the di-EMFs. In the 1200–1600 cm^−1^ domain, the most intense spectral lines of scandium mono- and di-EMFs are observed. In general, due to its higher symmetry (compared to that of mono-EMF), the di-EMF has fewer IR-active lines in all parts of the spectrum. The spectra of mono- and di-EMFs differ most significantly in the region below 400 cm^−1^ (the spectral domain corresponding to vibrations involving the metal atoms), see Figure 2, Figure 6, Figure 8 and Figure 9.

#### 2.7.2. Spectra of Yttrium Mono- and di-EMFs

In the range up to 400 cm^−1^, the Y@C_60_ mono-EMF shows one intense line at 53 cm^−1^ (lateral M–cage mode). For the di-EMF, Y_2_@C_60_ ($C_{3v}$), there is also only one intense line in this range at 246 cm^−1^ (longitudinal M–cage mode). In the 400–600 cm^−1^ domain, most of the vibration frequencies of the mono- and di-EMF differ slightly, with the exception of the 500–550 cm^−1^ interval, in which there are IR-active modes of the mono-EMF while the di-EMF has no IR-active lines of noticeable amplitude.

In the 800–1000 cm^−1^ range, the di-EMF has an IR line at 938 cm^−1^, but and the mono-EMF has no IR-active lines of noticeable amplitude. In the range 1000–1150 cm^−1^, there are IR-active mono-EMF’s modes but no noticeable IR-active lines of the di-EMF. This is due to the reduced symmetry of the mono-EMF. The most intense spectral lines of the mono- and di-EMF are observed in the frequency range 1200–1600 cm^−1^.

In the spectra of yttrium mono- and di-EMFs, many of the vibrational modes are very close in frequency, since metal atoms are involved in these vibrations (for example, IR-active lines 1175 cm^−1^ for Y@C_60_ and 1177 cm^−1^ for Y_2_@C_60_).

In general, due to higher (compared to the mono-EMF) symmetry, the di-EMF spectrum has fewer IR-active lines in all parts of the spectrum, with the spectrum differing most significantly in the region below 400 cm^−1^ (the domain corresponding to the of vibrations that involve the metal atom), see Figure 3, Figure 7 and Figure 10.

#### 2.7.3. Spectra of Sc and Y di-EMFs

The difference in the position of the most intense lines in the infrared spectra of M_2_@C_60_ from the IR-active lines of C_60_ is explained by a frequency shift due to the metal–carbon bond formation, as well as by the appearance of a number of IR-active lines that are symmetry-forbidden in the empty C_60_. In general, due to the lower symmetry of the Sc_2_@C_60_ ($C_{s}$) molecule, its spectrum contains more IR-active lines compared to the spectra of M_2_@C_60_ with $C_{3v}$ symmetry.

In the range up to 300 cm^−1^ in the IR spectrum of Sc_2_@C_60_ ($C_{s}$), there are no intense lines, for both EMFs with $C_{3v}$ symmetry; only one intense line is observed (longitudinal M–cage mode). Its frequency is 286 cm^−1^ for Sc_2_@C_60_ ($C_{3v}$) and 246 cm^−1^ for Y_2_@C_60_ ($C_{3v}$). The decrease in frequency can be explained by an increase in the atomic mass of the metal participating in the vibrations. In the 300–400 cm^−1^ spectral domain, a group of lines with frequencies 340–400 cm^−1^ is observed for Sc_2_@C_60_ ($C_{3v}$). For Sc_2_@C_60_ ($C_{s}$), a single intense line with a frequency of 319 cm^−1^ is observed, and there are no intense Y_2_@C_60_ ($C_{3v}$) IR lines in this range.

In the frequency range 400–600 cm^−1^ for both EMFs with $C_{3v}$ symmetry, the most intense lines are very close in frequency: 440 cm^−1^and 574 cm^−1^for Sc_2_@C_60_ ($C_{3v}$), 431 cm^−1^and 573 cm^−1^for Y_2_@C_60_ ($C_{3v}$). In this range, the spectrum of Sc_2_@C_60_ ($C_{s}$) also has close lines at 426 cm^−1^ and 576 cm^−1^, but the most intense are the 479 cm^−1^ and 505 cm^−1^ lines, which are absent in the spectra of M_2_@C_60_ with $C_{3v}$ symmetry. In the range 600–800 cm^−1^, a significant number of weak IR lines are observed for all M_2_@C_60_ under consideration. The strongest lines in this range are 622 cm^−1^ for Sc_2_@C_60_ ($C_{3v}$), a doublet at 604 cm^−1^ for Sc_2_@C_60_ ($C_{s}$), and 635 cm^−1^ for Y_2_@C_60_ ($C_{3v}$). In the range from 800 to 1000 cm^−1^, there is an IR line at 938 cm^−1^of yttrium di-EMF only; no noticeable IR lines are found for both isomers of scandium di-EMF.

Near the frequency 1182 cm^−1^ of the IR-active line of empty C_60_ (marked with an arrow on the spectrum), an intense line is observed in the spectra of all considered M_2_@C_60_ di-EMFs. The metal atoms do not participate in these vibrations and have a weak effect on the parameters of this line.

**Figure 11:**
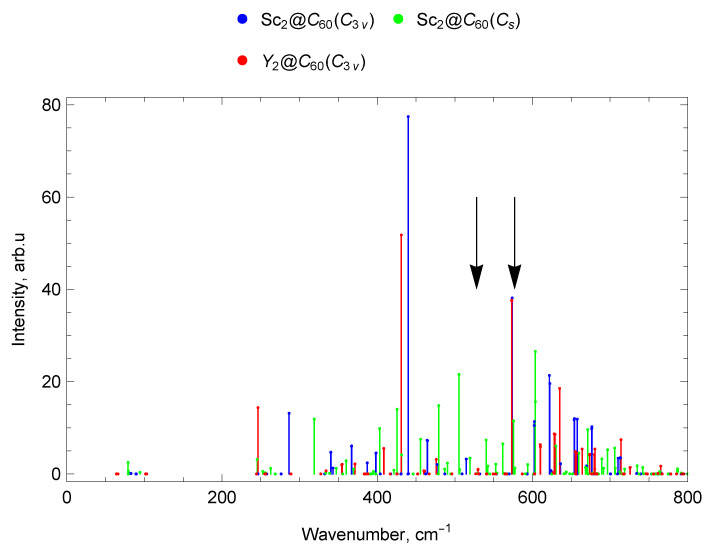
Comparison of IR spectra of Sc_2_@C_60_ ($C_{s}$), Sc_2_@C_60_ ($C_{3v}$) and Y_2_@C_60_ ($C_{3v}$) di-EMFs. The black arrows show the positions of two IR lines of the empty C_60_ fullerene ($\nu_{1}=527$ cm^−1^and $\nu_{2}=576$ cm^−1^ [62]).

The most intense lines of the IR spectra for all considered M_2_@C_60_ are located in the frequency range 1400–1500 cm^−1^. These lines correspond both to the IR-active line of empty C_60_ at 1429 cm^−1^ (which manifests in the IR spectra of EMFs), and to vibrational EMF modes that are IR-inactive for empty C_60_ fullerene; see Figure 8, Figure 9, Figure 10 and Figure 11.

Figure 11 compares the IR spectra below 800 cm^−1^ of the di-EMFs Sc_2_@C_60_ ($C_{s}$), Sc_2_@C_60_ ($C_{3v}$) and Y_2_@C_60_ ($C_{3v}$). The black arrows show the positions of two IR lines of the empty C_60_ fullerene ($\nu_{1}=527$ cm^−1^and $\nu_{2}=576$ cm^−1^ [62]).

## 3. Methods

DFT calculation of the scandium subgroup EMFs were performed in Gaussian-09 [69] using the hybrid mPW3PBE function [70]. All the atoms were described by the all-electron valence jorge-DZP basis set [71]. The correspondence of the calculated structures to the potential energy surface minimum was checked by the absence of imaginary vibration frequencies.

To confirm the efficiency of the computational scheme, we perform some preliminary calculations of structural, spectral, and thermodynamic characteristics for C_60_ fullerene and metal carbides MC_2_ of the scandium subgroup (M = Sc, Y). For these carbide molecules, our calculations reproduce their isosceles triangle geometry known from experiment, the bond lengths being in good agreement with experimental data. These preliminary calculations allow us to evaluate the accuracy of the chosen quantum-chemical model of aromatic multi-atomic systems involving heavy atoms, to which the considered EMFs belong. Table 2 compares the results of our calculations with the experimental values of atomization enthalpies $\Delta H^{\circ }$ (at 0 K) and bond lengths [72,73,74,75], as well as the vibrational frequencies $\nu_{1}$–$\nu_{3}$ calculated in Refs. [76,77].

The next step in the verification of the computational scheme was to test it on the “empty” C_60_ fullerene calculations. Table 3 compares compares the results of our calculations with the experimental values standard enthalpy of formation, $\Delta H^{\circ }$ ([78], pp. 5–9), and bond lengths, $r_{n–m}$ (length of the common edge of *n*- and *m*-membered rings in C_60_ fullerene) [79], as well as the vibrational frequencies $\nu_{1}$–$\nu_{4}$ measured in Ref. [62].

Because Sc and Y proton numbers are odd, the mono-EMFs M@C_60_ systems have open electronic shells, the same is the case for metal carbides, MC_2_. We used unrestricted Hartree–Fock (UHF) description of these systems. The ground electronic states of the mono-EMFs M@C_60_ are doublets (multiplicity M=2), while the di-EMFs M_2_@C_60_ are characterized by singlet ground states (M=1).

## 4. Conclusions

Endohedral metallofullerenes (EMFs) are prospective materials with a number of possible applications in optoelectronics, spintronics, medicine and quantum computations. In the present work, we make DFT calculations of stable structures and IR spectra of Sc@C_60_ and Y@C_60_ monometallofullerenes (mono-EMFs), as well as those of Sc_2_@C_60_ and Y_2_@C_60_ dimetallofullerenes (di-EMFs). For the mono-EMFs, Sc@C_60_ and Y@C_60_, the lowest stable state corresponds to the isomer with the $C_{s}$ symmetry. The lowest stable states of di-EMFs, Sc_2_@C_60_ and Y_2_@C_60_, correspond to the $C_{3v}$ symmetry isomer. However, we detected another stable state of Sc_2_@C_60_ isomer with $C_{s}$ symmetry which has the energy by 0.11 eV greater than that of the $C_{3v}$ isomer.

Thus, the structures under our study were Sc@C_60_ $\left(C_{s}\right)$, Y@C_60_$\left(C_{s}\right)$, Sc_2_@C_60_$\left(C_{3v}\right)$, Sc_2_@C_60_$\left(C_{s}\right)$ and Y_2_@C_60_$\left(C_{3v}\right)$. We calculated the IR vibration spectra of these EMFs. For all the EMFs studied, the IR spectra consist of many lines, most of which do not appear in the IR spectra of empty C_60_ fullerene because the corresponding transitions are symmetry-forbidden.

For the spectra of mono- and di-EMFs of scandium and yttrium, we describe the metal–cage modes (i.e., the molecular vibrations involved the metal atoms). These are longitudinal and transverse modes, as well as rotational modes of the metal dimer. We also determine the directions of atomic motion for metal–cage modes and indicate the symmetry features of atomic displacements for both IR-active and IR-inactive vibrations. The corresponding illustrations and animations (short movies) are presented in the Supplementary Materials. Among the vibrations involving metal atoms, longitudinal vibrations along the metal–metal bond are characterized by the highest intensity in the IR spectrum of M_2_@C_60_ due to the displacement of the positive charge center.

We make a comparison between the vibrational modes of mono- and di-EMFs. Some IR-active modes of mono-EMFs become IR-inactive in di-EMFs. For example, the pumpkin vibrational mode in Sc@C_60_ and Y@C_60_, which is associated with simultaneous stretching-compression of all metal–carbon bonds, is IR-active. In the Sc_2_@C_60_ and Y_2_@C_60_ di-EMFs, similar modes are associated with symmetric compression–stretching of the metal dimer without displacement of the center of positive charge, so these oscillations are not IR-active.

We show that some Raman and “silent” modes of the empty C_60_ fullerene become IR-active in M@C_60_ and M_2_@C_60_ EMFs due to the influence of the encapsulated species.

In the EMF synthesis, the Sc_2_@C_60_ and Y_2_@C_60_ molecules have not been extracted experimentally to date, so the obtained IR spectra could help to detect and identify these EMFs.

## Figures and Tables

**Table 1:** Interatomic distances in the calculated EMF structures.

	${\mathrm{Sc@C}}_{60}$ ($C_{s}$)	${\mathrm{Y@C}}_{60}$ ($C_{s}$)	${\mathrm{Sc}}_{2}{\mathrm{@C}}_{60}$ ($C_{3v}$)	${\mathrm{Sc}}_{2}{\mathrm{@C}}_{60}$ ($C_{s}$)	$Y_{2}{\mathrm{@C}}_{60}$ ($C_{3v}$)
*r*(M–C_1_), Å	2.17	2.34	2.26	2.24	2.36
*r*(M–C_2_), Å	2.24	2.40	2.26	2.24	2.36
*r*(M–C_3_), Å	2.33	2.48	2.26	2.25	2.36
*r*(M–M), Å	–	–	3.25	3.11	3.13

**Table 2:** The calculated (this work) and experimental characteristics of metal carbides.

	${\mathrm{ScC}}_{2}$		${\mathrm{YC}}_{2}$	
	calc.	expt.	calc.	expt.
$\Delta H^{\circ }$, kJ/mol	1183	1182	1239	1254
*r*(M–C), Å	2.046	2.057	2.191	2.187
*r*(C–C), Å	1.278	1.259	1.277	1.270
$\nu_{1}$, cm^−1^	335	330.0	337	342.0
$\nu_{2}$, cm^−1^	662	634.6	604	581.1
$\nu_{3}$, cm^−1^	1816	1767.9	1826	1839.7

**Table 3:** The calculated (this work) and experimental characteristics of the “empty” C_60_ fullerene.

	calc.	expt.	diff.
$\Delta H^{\circ }$, kJ/mol	2405	2502	4.2%
$r_{5–6}$, Å	1.451	1.452	0.1%
$r_{6–6}$, Å	1.397	1.397	<0.1%
$\nu_{1}$, cm^−1^	523	527	0.76%
$\nu_{2}$, cm^−1^	595	576	3.2%
$\nu_{3}$, cm^−1^	1233	1182	4.1%
$\nu_{4}$, cm^−1^	1497	1429	4.6%

## Data Availability

The data are available from the authors.

## Supplementary Materials

The following supporting information can be downloaded at: https://www.mdpi.com/article/10.3390/molecules30163421/s1.

## Appendix A

**Table A1:** Vibration frequencies ($\nu_{k}$, cm^−1^) and line intensities ($I_{k}$, relative units.) for M@C_60_ endofullerenes.

No.	${\mathrm{Sc@C}}_{60}$ ($C_{s}$)		${\mathrm{Y@C}}_{60}$ ($C_{s}$)	
	$\nu_{k}$	$I_{k}$	$\nu_{k}$	$I_{k}$
1	54.9188	10.5034	49.762	0.5939
2	84.3682	1.3061	52.622	5.1854
3	231.9072	2.1295	203.7065	2.1403
4	245.2244	1.8977	236.669	2.3121
5	254.8434	0.275	254.2988	0.1129
6	265.6296	0.036	262.8707	0.0603
7	269.5616	0.37	266.813	0.1857
8	319.6795	1.2952	304.1148	0.5722
9	335.255	1.2511	331.6442	2.7561
10	338.5788	0.9032	340.8941	1.3791
11	344.0158	1.3401	342.1575	1.0795
12	345.6743	0.0243	344.2537	0.0151
13	354.3939	1.5238	351.5146	0.5949
14	364.2819	0.1483	363.0392	0.0128
15	378.7477	0.8108	365.9182	0.0229
16	398.3175	0.78	394.3682	1.7211
17	399.396	0.0095	395.6482	0.4245
18	399.772	0.6038	396.2064	1.315
19	401.7526	0.2977	398.8513	0.7967
20	404.719	0.3872	400.7425	0.1307
21	416.5978	2.3301	404.1454	0.5953
22	422.8969	1.1861	418.6619	0.9549
23	426.2233	11.2429	421.8858	15.0228
24	426.9951	2.4991	424.7635	0.0654
25	442.7812	3.82	437.2006	3.3695
26	463.2727	2.3697	461.6701	0.6099
27	479.0408	0.0182	478.956	0.2597
28	484.8858	2.7619	484.2166	1.3652
29	493.4576	0.8953	488.6431	1.863
30	500.0551	0.0035	492.2553	4.6305
31	500.9249	1.8808	493.8308	0.786
32	506.4679	3.1632	505.055	2.8607
33	511.6815	1.0673	510.2221	1.2842
34	519.3214	10.2078	516.4876	6.8183
35	523.8925	0.8604	522.3363	0.2304
36	529.5547	10.6873	524.3846	5.0813
37	538.0455	0.0683	532.4612	0.0058
38	543.0311	0.5084	542.3137	0.3999
39	552.3908	1.145	548.5767	1.2618
40	553.0114	0.0223	552.3477	0.0395
41	555.042	0.3994	554.0949	0.3178
42	557.2458	2.8281	554.637	0.8385
43	563.5956	2.3317	564.7591	0.9204
44	565.9737	0.1446	564.9387	0.03
45	571.9696	0.0044	571.3255	0.02
46	575.3572	4.6815	572.7128	1.9636
47	578.9891	2.1215	578.4874	10.0132
48	583.4793	15.3897	578.5398	1.5721
49	592.3889	26.0894	591.3812	30.938
50	606.2789	12.8187	598.0936	19.172
51	609.53	0.7846	603.9177	2.4704
52	637.2138	2.3095	618.081	19.1911
53	640.9119	1.9561	629.5912	1.8307
54	651.6078	1.1549	650.2402	0.6928
55	662.381	0.0207	659.5293	0.256
56	663.3835	1.9392	661.6663	3.7551
57	666.241	0.1712	664.5723	0.3112
58	667.1808	0.3004	665.2914	0.004
59	668.799	3.6108	668.0754	4.4051
60	670.3037	2.6129	671.0978	1.0753
61	674.1803	0.0278	671.7388	2.1576
62	676.6796	4.4683	678.7048	0.0388
63	677.0959	1.0939	678.7211	3.099
64	683.0884	1.2403	683.9271	0.5006
65	687.6209	11.1933	686.1794	17.0234
66	688.7571	0.8989	689.2218	1.1416
67	691.4907	4.1649	690.6114	2.7627
68	693.3245	1.6307	692.8733	3.6523
69	694.0059	1.4743	695.2835	1.5051
70	695.173	2.2695	695.9895	2.2944
71	704.101	0.0227	701.9196	0.2835
72	704.7625	0.494	703.439	1.4335
73	705.0558	0.3317	703.9615	0.2426
74	705.0664	0.0904	703.9766	0.3184
75	709.009	0.1873	707.747	0.011
76	711.8809	0.087	712.2943	0.3092
77	722.2866	3.2397	720.3689	2.4373
78	727.7552	0.1452	727.1894	0.031
79	736.5369	1.9579	736.1791	3.6256
80	738.2888	0.1788	750.9225	0.0559
81	755.761	0.0073	756.5885	0.0462
82	758.2315	0.0831	759.3982	0.835
83	760.5557	0.3114	760.0718	0.0453
84	762.3751	0.0989	761.4314	0.0444
85	778.562	4.9404	775.3487	6.5901
86	783.8644	1.1869	781.1952	2.1291
87	788.0201	0.1788	782.9962	0.4141
88	788.6326	0.0309	787.9893	0.0814
89	799.8413	0.168	793.9862	0.1776
90	801.5983	0.2498	799.1997	0.1369
91	803.2158	0.239	802.6487	0.2448
92	803.2443	0.3458	802.6651	0.0862
93	825.739	0.1767	824.7525	0.1069
94	826.8086	0.1544	826.1007	0.0672
95	829.581	0.0003	827.9333	0.0032
96	941.4669	0	938.9152	0.0013
97	973.1506	1.3964	970.1702	3.52
98	975.1995	1.242	973.309	0.9045
99	982.0183	1.0759	978.9959	1.0072
100	984.5874	0.6413	981.6339	0.6234
101	1001.0734	6.3485	1000.9679	10.0023
102	1005.9538	0.6753	1004.6588	1.3841
103	1010.1159	2.2504	1010.3693	2.1099
104	1069.2678	19.8583	1050.2398	14.0723
105	1104.8915	8.3598	1094.5464	18.5848
106	1106.6563	3.6433	1097.6029	5.0212
107	1121.624	4.2392	1117.9292	3.8061
108	1131.0334	2.6283	1128.2198	3.4907
109	1134.3259	0.0941	1134.1728	0.55
110	1144.1817	1.6187	1142.0874	2.9744
111	1144.2033	3.6899	1143.1169	1.9164
112	1144.889	0.3629	1144.7654	0.7733
113	1181.7175	9.4999	1175.1109	7.5938
114	1203.9204	6.4762	1195.3264	10.1651
115	1211.0441	2.7927	1203.1612	0.911
116	1212.4373	11.7736	1204.1803	13.5954
117	1224.9623	6.3276	1222.5103	6.9472
118	1231.1092	0.4618	1228.9144	7.3897
119	1233.9767	0.9676	1229.4857	3.0721
120	1236.8193	7.9966	1234.8869	5.6621
121	1240.1197	14.7899	1236.7402	18.5959
122	1241.8546	1.1859	1238.9986	3.7997
123	1252.3547	1.0543	1249.4936	3.4864
124	1275.8072	3.3947	1266.1577	8.0192
125	1285.4383	0.7219	1277.75	2.9424
126	1293.4739	5.8009	1287.7458	8.2785
127	1298.0086	5.622	1296.5271	6.7523
128	1306.0515	1.0948	1302.3494	2.4795
129	1307.2429	0.6915	1305.7566	0.7031
130	1309.6005	3.1042	1306.104	4.7755
131	1322.3785	0.0002	1321.0631	0.0612
132	1331.2151	3.0344	1328.4262	2.5652
133	1341.777	0.2741	1336.0932	0.9261
134	1345.581	1.5801	1343.9778	1.7768
135	1355.2006	0.3488	1347.2503	3.1169
136	1356.7307	1.1597	1353.1607	0.6969
137	1359.6725	0.1918	1357.4534	0.1664
138	1362.4249	1.3346	1360.0586	1.8072
139	1365.4416	0.1414	1362.5424	0.0419
140	1365.9558	0.2896	1363.3912	2.7744
141	1373.0613	3.5375	1367.8749	2.2178
142	1373.8142	0.7722	1371.9478	9.1227
143	1379.0199	1.5245	1375.033	0.845
144	1386.1731	0.0147	1379.1069	0.0336
145	1388.7594	6.1198	1384.0419	26.5846
146	1389.8179	20.8553	1385.4215	38.9756
147	1396.4684	1.0623	1391.6847	68.3
148	1398.8875	11.5202	1395.6489	11.8558
149	1402.7378	22.337	1396.4134	27.749
150	1411.8222	74.3599	1399.8106	38.3463
151	1433.885	74.3562	1420.3688	79.1329
152	1449.0511	28.2437	1435.8272	44.7941
153	1456.4103	11.3805	1447.0131	95.5718
154	1458.6693	68.3782	1455.2032	86.6558
155	1467.1926	47.612	1458.1421	22.6936
156	1468.3445	1.7234	1468.2107	12.2601
157	1474.4349	27.5706	1471.7573	37.0593
158	1482.7782	8.8604	1479.7459	8.3834
159	1484.6975	27.6066	1482.0358	23.1165
160	1501.0501	6.3663	1495.6085	6.3091
161	1508.524	0.9038	1503.7295	1.0529
162	1519.3326	27.1187	1520.5333	21.5776
163	1539.6454	0.0009	1533.5779	5.6703
164	1542.481	5.6754	1534.495	0.9343
165	1544.5977	6.4449	1536.6477	6.2245
166	1549.4958	0.4941	1541.3156	1.2692
167	1551.6914	1.0817	1544.3037	4.0452
168	1553.4086	0.4043	1545.6009	0.2033
169	1563.0796	2.8773	1554.5661	1.1125
170	1571.1412	2.4778	1561.5705	2.1147
171	1572.8589	2.5782	1568.3456	2.6574
172	1583.0124	4.0122	1575.1561	4.2037
173	1598.0393	5.2015	1590.9557	8.5787
174	1601.3145	14.1149	1595.6297	23.3172
175	1604.6917	9.0311	1602.8863	8.3741
176	1611.2009	8.6319	1605.774	15.5851
177	1612.355	5.146	1607.8493	8.5419

**Table A2:** Vibration frequencies ($\nu_{k}$, cm^−1^) and line intensities ($I_{k}$, relative units.) for M_2_@C_60_ endofullerenes.

No.	${\mathrm{Sc}}_{2}{\mathrm{@C}}_{60}$ ($C_{3v}$)		${\mathrm{Sc}}_{2}{\mathrm{@C}}_{60}$ ($C_{s}$)		$Y_{2}{\mathrm{@C}}_{60}$ ($C_{3v}$)	
	$\nu_{k}$	$I_{k}$	$\nu_{k}$	$I_{k}$	$\nu_{k}$	$I_{k}$
1	81.95	0.0891	78.95	2.5033	63.95	0.0000
2	82.6828	0.0991	79.24	0.0050	65.99	0.0000
3	89.0201	0	79.39	0.5868	101.49	0.0028
4	89.6563	0	94.37	0.3545	102.91	0.0038
5	246.3521	0	245.22	3.1424	244.43	0.0001
6	257.2319	0	252.59	0.5416	246.34	14.3723
7	257.6413	0	255.78	0.2414	254.99	0.0000
8	276.0961	0.0002	262.64	1.1927	256.45	0.0000
9	276.2172	0.0001	268.56	0.0000	288.63	0.0001
10	286.4063	13.1518	318.83	11.8847	288.83	0.0000
11	332.2817	0.0017	333.69	0.1506	327.27	0.0005
12	340.0485	4.6591	338.53	0.1369	334.06	0.6695
13	340.2518	4.7108	347.14	1.2174	354.24	1.9465
14	342.9838	1.2709	355.53	0.0000	354.80	2.0762
15	366.8752	5.9916	359.91	2.8679	371.23	2.1276
16	366.971	6.0703	369.47	1.0545	371.30	2.1624
17	386.998	2.3818	386.84	0.0332	383.21	0.0017
18	387.1514	2.4051	390.96	0.0000	383.34	0.0003
19	391.5877	0.0028	393.21	0.0315	385.23	0.0000
20	398.1662	4.4883	395.12	0.5279	388.18	0.0001
21	398.5032	4.5393	398.01	0.0000	408.35	5.5303
22	403.912	0.0002	403.06	9.8448	408.56	5.5210
23	417.3545	0	421.45	0.8017	416.58	0.0001
24	430.3878	0	425.30	0.1248	425.18	0.0004
25	430.7603	0	425.52	14.0096	425.68	0.0000
26	439.518	0.0001	431.30	4.1123	431.05	51.7812
27	439.882	77.4321	446.19	0.0000	451.87	0.0000
28	440.1611	0.0555	455.84	7.5080	452.28	0.0001
29	462.2293	0.0002	478.68	0.0031	460.01	0.6850
30	464.2559	7.295	479.20	14.8063	460.99	0.6246
31	464.7875	7.2196	486.83	1.0518	466.97	0.0000
32	477.0306	2.0317	490.38	2.3426	476.07	3.1430
33	486.9704	0	494.67	0.0000	496.17	0.0000
34	509.3085	0.0074	505.36	21.5666	526.88	0.0260
35	514.5194	3.2183	506.19	0.9547	529.70	0.9291
36	514.787	3.2154	519.52	3.4110	529.94	0.9939
37	531.1489	0	528.75	0.0927	530.64	0.0078
38	531.4358	0	540.27	7.3260	540.64	0.0000
39	532.068	0	542.38	1.6787	541.14	0.0000
40	549.7759	0	545.00	0.0000	549.30	0.0000
41	553.6764	0	549.92	0.0000	552.54	0.0001
42	553.7494	0	552.69	2.0966	552.64	0.0000
43	564.5495	0	554.21	0.1566	564.45	0.0002
44	564.6892	0	561.76	6.5229	565.45	0.0000
45	568.1453	0	563.62	0.1104	566.35	0.0003
46	570.0265	0.0015	567.02	0.0000	566.44	0.0002
47	573.756	38.1059	575.60	11.4872	573.18	37.6595
48	573.8159	38.1402	577.37	1.2664	573.28	37.5027
49	592.5356	0.0002	590.65	0.0219	586.86	0.0002
50	592.7212	0.0001	593.83	2.0420	602.52	0.0014
51	593.1775	0.0008	601.53	0.0107	602.90	0.0000
52	602.234	10.492	603.66	15.6539	609.98	6.3385
53	602.4031	11.3363	603.69	26.5221	610.24	5.8881
54	621.7374	21.346	624.80	0.0000	627.05	0.3042
55	622.3506	19.5939	630.13	6.0526	628.03	8.6707
56	623.9753	0.7065	640.48	0.0340	629.03	8.5735
57	636.1944	2.2156	643.48	0.4214	634.98	18.5265
58	653.6652	11.7356	647.90	0.0361	655.08	4.8152
59	654.105	11.9753	651.53	0.0000	655.41	0.0195
60	656.8444	0.0006	659.55	4.5040	655.95	0.0007
61	657.3784	0.0061	668.31	1.4273	656.75	4.4998
62	657.6973	11.8607	670.18	1.1774	663.97	5.3958
63	669.6253	1.7236	671.20	9.5921	673.65	4.0864
64	669.8302	1.6076	671.53	0.0003	673.81	4.2600
65	676.2943	9.7942	676.35	0.0295	676.38	0.0002
66	676.5571	10.223	676.94	0.0000	679.32	0.0718
67	676.7821	0.0021	677.70	0.2084	679.65	4.2042
68	677.3025	0.0013	683.31	0.0944	680.12	1.0623
69	677.6551	0.0271	684.95	0.0000	680.39	5.4029
70	678.2954	0.0001	689.37	3.2574	681.99	0.0059
71	680.814	0	691.51	1.2116	683.15	0.0003
72	700.2868	0	696.74	5.2250	714.04	7.4142
73	700.5939	0	705.86	5.5792	717.12	0.0017
74	709.5648	0.0001	706.51	1.2426	717.52	0.0024
75	710.3009	3.3806	712.45	0.4658	718.24	0.0002
76	713.0645	3.5423	716.04	0.0000	725.54	1.3798
77	713.4601	3.4636	718.69	1.0675	725.91	1.3945
78	734.1041	0.094	734.82	1.7653	746.32	0.0250
79	734.3033	0.1076	737.29	0.0000	746.41	0.0203
80	739.3554	0	742.02	1.3988	748.03	0.0002
81	739.498	0	743.59	0.0334	755.77	0.0000
82	753.1752	0.0003	753.61	0.0246	755.97	0.0000
83	753.2007	0	758.02	0.0011	761.56	0.0931
84	758.9885	0	760.67	0.0000	761.76	0.0957
85	761.4512	0.0005	763.46	0.0000	765.27	1.6817
86	761.5185	0.0006	764.00	0.5207	766.74	0.0008
87	766.0023	0.1636	764.66	0.0055	767.10	0.0001
88	775.852	0	774.78	0.0000	778.28	0.0004
89	776.0167	0	786.86	1.0939	785.52	0.0000
90	791.457	0	786.91	0.5732	792.36	0.0000
91	791.6218	0	791.72	0.1765	792.47	0.0000
92	800.0554	0	799.88	0.0000	794.11	0.0001
93	805.6614	0	804.42	0.1150	794.86	0.0000
94	805.8526	0	805.47	0.2774	802.17	0.0000
95	808.051	0	805.49	0.0256	802.24	0.0000
96	825.6711	0	824.75	0.0196	824.02	0.0000
97	825.7765	0	824.97	0.0000	824.13	0.0001
98	831.5413	0	830.38	0.0145	826.71	0.0000
99	940.985	0.0001	940.96	0.0000	935.18	0.0055
100	960.6919	3.0671	962.80	4.0195	938.13	7.5552
101	970.0638	0.0169	963.32	2.0807	956.24	0.2956
102	970.1174	0.015	970.33	0.0000	956.56	0.2961
103	983.2568	0.0001	982.16	0.0141	981.95	0.0001
104	1014.251	1.0647	1011.96	0.8107	1001.44	0.0430
105	1014.487	1.0751	1013.89	0.0286	1001.55	0.0417
106	1020.997	0.3986	1017.28	0.1740	1015.30	0.0021
107	1110.588	0	1105.21	0.0000	1093.94	0.0000
108	1110.621	0	1105.31	0.1068	1094.02	0.0001
109	1123.244	0	1118.83	0.0550	1100.79	0.0000
110	1123.404	0	1120.57	4.9126	1100.90	0.0002
111	1128.135	0	1130.58	0.2243	1103.17	0.0000
112	1151.748	0.0002	1138.20	3.9136	1108.10	0.0970
113	1152.372	8.6974	1156.04	0.0000	1108.66	0.0907
114	1152.537	8.6067	1156.62	2.3323	1137.65	0.0003
115	1163.412	0	1157.63	0.1048	1155.21	0.0002
116	1163.468	0	1159.73	0.0000	1155.31	0.0001
117	1165.1	0	1169.09	4.2220	1156.67	0.0000
118	1186.293	71.0026	1202.70	11.4163	1177.45	26.9316
119	1186.438	70.8642	1204.63	17.9355	1177.51	27.1322
120	1213.316	0.0012	1216.64	5.7063	1198.44	0.5152
121	1219.581	4.42	1223.06	8.6008	1198.57	0.3940
122	1219.821	4.4907	1223.33	2.0393	1201.28	8.2368
123	1226.307	7.0833	1223.39	0.0003	1206.87	0.0006
124	1245.295	0.0011	1238.88	0.4244	1225.29	0.0001
125	1245.421	0.0013	1250.03	4.0419	1225.48	0.0004
126	1259.181	0.0001	1253.01	1.7399	1243.67	0.6242
127	1259.237	0	1258.45	5.8822	1243.73	0.6234
128	1262.828	1.6848	1275.01	12.2292	1247.57	5.8973
129	1298.759	0	1275.16	0.0000	1277.21	0.0002
130	1298.822	0	1294.82	0.4487	1277.34	0.0009
131	1302.634	0	1307.98	0.5697	1284.88	0.0011
132	1318.451	0	1313.54	0.5133	1305.02	0.0027
133	1318.577	0	1314.33	0.0000	1312.35	0.0058
134	1333.39	0	1324.82	1.9123	1312.68	0.0077
135	1341.82	18.4106	1337.41	0.0000	1315.44	177.9984
136	1341.877	18.4887	1348.65	0.0000	1317.54	23.9301
137	1350.26	0	1349.83	17.6868	1317.92	25.0840
138	1361.42	0.0045	1354.19	6.7394	1331.82	0.0089
139	1363.96	0	1355.74	0.4737	1340.77	0.0082
140	1363.971	0	1358.22	6.1220	1346.10	0.0121
141	1365.289	0.0001	1364.80	0.0434	1346.79	81.2490
142	1367.395	36.2336	1370.87	0.0010	1347.23	82.6081
143	1370.049	1.8206	1374.98	0.0000	1349.60	0.0025
144	1370.413	1.9469	1376.59	21.4997	1349.86	0.0208
145	1376.264	0.0023	1381.82	12.2208	1364.25	10.6934
146	1386.941	13.1141	1385.77	25.6910	1364.41	10.9053
147	1387.036	14.325	1387.02	0.0046	1365.19	0.0058
148	1390.861	0.0002	1388.85	48.5261	1365.28	0.0138
149	1390.993	0.0001	1392.17	0.0763	1371.40	0.0078
150	1402.414	0.0007	1395.89	53.7825	1372.93	22.0939
151	1402.654	185.532	1398.46	0.0000	1373.17	84.6566
152	1403.807	277.0629	1404.65	15.0140	1384.21	53.7793
153	1403.904	279.1288	1413.42	35.3228	1384.33	54.4713
154	1417.706	0.0051	1413.73	85.3519	1393.55	0.0000
155	1425.242	0.0004	1414.49	142.6477	1397.62	0.0007
156	1425.284	0.0009	1416.01	0.0136	1397.80	0.0010
157	1451.926	192.5961	1448.29	108.8656	1428.93	326.6849
158	1452.03	193.8582	1448.37	1.1858	1428.99	325.9180
159	1460.183	0	1454.97	32.7941	1446.09	0.0006
160	1460.246	0	1455.17	0.0850	1446.11	0.0003
161	1462.182	0.0292	1465.19	1.8584	1451.21	0.0085
162	1470.152	14.083	1468.47	45.2911	1455.82	31.2813
163	1485.167	0.3396	1480.60	0.0367	1466.23	0.0005
164	1485.246	0.3957	1483.05	19.2811	1466.46	0.0005
165	1490.299	0	1488.98	0.0000	1476.84	0.2428
166	1490.433	0.0001	1509.09	16.2044	1476.98	0.2008
167	1509.153	0	1512.26	6.0089	1502.95	0.0001
168	1523.09	17.2113	1518.62	0.4619	1505.30	16.6201
169	1523.397	17.4556	1532.34	0.3468	1505.51	16.4199
170	1527.187	18.1574	1533.65	0.0000	1515.71	0.0000
171	1527.337	17.4534	1534.18	0.0326	1521.41	5.8033
172	1529.74	0.0003	1538.52	0.0000	1521.60	5.8307
173	1534.508	0	1545.24	1.5445	1521.65	0.0559
174	1542.936	0.0014	1547.81	0.4148	1526.44	1.9181
175	1548.994	0.6798	1558.62	0.7472	1536.47	0.0000
176	1550.791	0	1559.33	0.3628	1536.72	0.0000
177	1550.89	0	1569.51	2.1475	1538.09	0.0005
178	1573.825	0	1576.79	0.0001	1559.16	0.0000
179	1573.97	0	1577.46	1.6381	1559.34	0.0000
180	1578.164	0	1592.76	0.6369	1576.68	0.0001
